# Using DNA barcoding to improve invasive pest identification at U.S. ports-of-entry

**DOI:** 10.1371/journal.pone.0222291

**Published:** 2019-09-17

**Authors:** Mary J. L. Madden, Robert G. Young, John W. Brown, Scott E. Miller, Andrew J. Frewin, Robert H. Hanner

**Affiliations:** 1 Department of Integrated Biology, University of Guelph, Guelph, Ontario, Canada; 2 Entomology Department, National Museum of Natural History, Smithsonian Institution, Washington, D.C., United States of America; Universita degli Studi di Milano-Bicocca, ITALY

## Abstract

Interception of potential invasive species at ports-of-entry is essential for effective biosecurity and biosurveillance programs. However, taxonomic assessment of the immature stages of most arthropods is challenging; characters for identification are often dependent on adult morphology and reproductive structures. This study aims to strengthen the identification of such specimens through DNA barcoding, with a focus on microlepidoptera. A sample of 241 primarily immature microlepidoptera specimens intercepted at U.S. ports-of-entry from 2007 to 2011 were selected for analysis. From this sample, 201 COI-5P sequences were generated and analyzed for concordance between morphology-based and DNA-based identifications. The retrospective analysis of the data over 10 years (2009 to 2019) using the Barcode of Life Data (BOLD) system demonstrates the importance of establishing and growing DNA barcode reference libraries for use in specimen identification. Additionally, analysis of specimen identification using public data (43.3% specimens identified) vs. non-public data (78.6% specimens identified) highlights the need to encourage researchers to make data publicly accessible. DNA barcoding surpassed morphological identification with 42.3% (public) and 66.7% (non-public) of the sampled specimens achieving a species-level identification, compared to 38.3% species-level identification by morphology. Whilst DNA barcoding was not able to identify all specimens in our dataset, its incorporation into border security programs as an adjunct to morphological identification can provide secondary lines of evidence and lower taxonomic resolution in many cases. Furthermore, with increased globalization, database records need to be clearly annotated for suspected specimen origin versus interception location.

## Introduction

Invasive insects are of global concern, causing long-term environmental impacts including reduced ecosystem stability and loss of native species [1]. Direct damage to commodities and management activities focused on invasive insect pests cost the global economy billions of dollars each year [2–4]. With the ever-increasing global movement of commodities and intensifying effects of global warming, there is little doubt that the frequency of insect invasions will increase [5–8]. To reduce the establishment of invasive species, border inspection programs intercept and identify potential invasive pests and are an integral part of effective biosecurity policies. Data from border inspection programs inform policies regarding import restrictions and guidelines and can trigger biosecurity actions such as trade sanctions and commodity decontamination [9,10].

Border inspection data can also be used in pathway analysis of invasive species, increasing regulators’ ability to understand common routes of insect invasions [10]. However, Kenis *et al*. 2007 [11] concluded that only 10% of the invasive species with confirmed populations in non-native ranges were detected at ports-of-entry prior to their establishment, clearly demonstrating a need to improve biosecurity and biosurveillance policies. Strategies to identify introduction pathways through specimen detection and identification have been developed for several countries including the United Kingdom [12,13], Australia [14], and Finland [15]. Ultimately, the effectiveness of such programs relies on the ability to intercept specimens in transit and quickly identify them to a taxonomic rank necessary to inform biosecurity actions and policies.

Microlepidoptera are a paraphyletic grade of Lepidoptera, generally described as ‘small moths’ [16]. Identifications of microlepidoptera from taxonomic ranks of family to species, relies on morphological characters of the adult, primarily those of the genitalia [17, 18]. Because most microlepidoptera are intercepted as larvae, they pose a special challenge for accurate and timely identifications [3, 19]. Although critical evaluations on the reproducibility of morphology-based taxon assignments are limited, subjective interpretations occur, resulting in inconsistent taxonomic assignment of specimens by different investigators, especially at lower taxonomic ranks [17, 20].

Many border-intercepted specimens are identified at higher ranks (e.g., family or subfamily) due to the challenges of classifying specimens of immature life stages [21]. This is illustrated by the fact that only 40% of the specimens in a USDA interception dataset, received species-level identifications. While taxonomic identifications above species are useful in some investigations, regulatory management of invasive biological organisms typically requires species-specific information such as life history, larval hosts, parasitoids and dispersal ability [22, 23].

A potential solution to the challenge of morphology-based identifications for border interception programs is the integration of DNA barcoding. DNA barcoding is a standardized molecular identification method with numerous applications that has been used extensively to identify immature life stages of animals [24–31]. The Barcode of Life Data Systems (BOLD) is a publicly accessible domain providing a reference library and analytical capabilities for DNA barcode projects [32]. One strength of the BOLD system is in the association of records with metadata including chromatogram files, the location of voucher specimens, and the presence and location of long-term storage of DNA or tissue [33, 34]. These data are only useful when they are publicly accessible to external users, allowing for verification of completeness and accuracy of records. Additionally, BOLD periodically mines sequences from GenBank, increasing the number of sequences available for data analysis [35, 36].

An important feature of the BOLD platform is the barcode index number (BIN). BINs are molecular operational taxonomic units [37] (MOTU) generated by the Refined Single Linkage (RESL) algorithm [38], based on available BOLD data. BINs provide interim taxonomic identifications or suspected species classification, based on a molecular barcode [38]. The BIN framework is most suitable for microlepidoptera identification as it has been shown to be in general agreement with morphology-based species-level identifications [38, 39]. Furthermore, when the RESL algorithm was being developed it was extensively tested against a large lepidoptera dataset [38]. The capability of DNA barcoding to aid in the identification of specimens can be measured by its success in providing taxonomic resolution equivalent to or better than that achieved by traditional methods. This can include, specimens that can be identified to the species level or specimens that are grouped into an interim taxonomic framework (i.e., BINs).

To test the use of DNA barcoding in the identification of insect specimens, we examined a set of predominantly immature microlepidoptera from the superfamilies Tortricoidea and Gelechioidea. Both Tortricoidea and Gelechioidea are diverse, containing numerous regulated and economically important species, many of which are represented in BOLD [25]. The first objective of this study is to examine the concordance between morphology-based and DNA-based identifications for intercepted microlepidoptera. The second objective is to develop a framework for the use of DNA barcoding and BIN interim taxonomy with respect to border identification protocols for intercepted insect specimens.

We obtained DNA barcodes from 201 microlepidoptera specimens intercepted by USDA Animal and Plant Health Inspection Service Plant Protection and Quarantine (USDA-APHIS-PPQ) inspectors at U.S. ports-of-entry and identified by the USDA Systematic Entomology Laboratory (USDA-SEL). Morphological identifications were then compared to the DNA barcode-based identifications and BIN assignments given to each specimen by BOLD. Over time, the number of specimens in the interception dataset matching a sequence in the BOLD database increased. However, a lack of data movement from private to public records was observed. Through these analyses we demonstrate a need for continued population of molecular libraries with good quality, public data to increase the strength of DNA barcode identifications.

## Materials and methods

### Sequencing

USDA APHIS-PPQ personnel routinely inspect incoming commodities at U.S. ports-of-entry (including Puerto Rico) for plant and animal pests. Unidentified insect specimens were preserved in 75% alcohol (immatures) or pinned (adults) and submitted to the USDA-SEL for identification. Interceptions from commercial commodities are labelled “urgent” and are sent by overnight courier to USDA-SEL for rapid turnaround (i.e., identification the same day the specimen(s) is received by USDA-SEL). Non-critical, non-commercial interceptions labelled “routines,” are saved at the port and periodically sent to USDA-SEL for evaluation.

We selected a sample of microlepidoptera specimens (241) intercepted by APHIS-PPQ consisting mainly of larvae, but with a few adult specimens. The specimens were identified by USDA-SEL staff located in the Entomology Department of the National Museum of Natural History at the Smithsonian Institution in Washington, DC. USDA-SEL staff rely on origin of interception, host plant or other association, and historical records to augment morphology when identifying samples. The collection was assembled with an effort to include a broad range of frequently intercepted microlepidopteran families: Tineidae, Blastobasidae, Cosmopterigidae, Gelechiidae, Oecophoridae, Tortricidae, Plutellidae, Pterophoridae, and Sesiidae, and a few specimens of unknown familial affinity. Nonetheless, Tortricioidea and Gelechioidea dominated the samples because larvae of these two superfamilies are the most commonly intercepted and submitted samples of microlepidoptera.

For adult specimens a single hind leg was used as a source of genomic DNA. For immature specimens (larvae or pupae) > 5 mm in length, DNA was obtained from a 2–3 mm^2^ piece of tissue removed from the lateral side of the specimen with flame-sterilized forceps and scissors, while specimen <5 mm in length had their entire bodies homogenized to extract DNA. Genomic DNA was extracted following the alkaline lysis DNA extraction XytXtract Insect (ANDE) (Xytogen; Perth, Australia) kit using manufacturer recommended protocols [40] and stored at -20°C prior to analysis.

All PCR reactions were conducted in a total volume of 12.5μL, as described by Wilson et al. (2012) [41] as modified from Ivanova et al. (2009) [42]. PCR amplification was completed using primer pairs LepF1, 5′-ATTCAACCAATCATAAAGATAT-3′; and LepR1, 5′-TAAACTTCTGGATGTCCAAAAA-3′ [43]. Reactions that failed to produce sufficient DNA products for sequencing underwent a second amplification using primer pairs mLepF1/LepR1, and LepF1/mLepR1 as described by Wilson et al. (2012) [41]. The following cycling conditions were used for all primer pairs: an initial denaturation at 94°C for 1min, followed by 5 cycles of 94°C for 40s, 45°C for 40s, 72°C for 1min, followed by 35 cycles of 94°C for 40s, 51°C for 40s, 72°C for 1min, and a final extension at 72°C for 5min [44]. PCR products were visualized on a 2% agarose gel pre-stained with SYBR® Safe DNA gel stain (Life Technologies^™^). PCR products were sent to the Advanced Analysis Centre at the University of Guelph (Guelph, Ontario, Canada) for sequencing. Sequencing was performed on an Applied Biosystems® 3730 DNA Analyzer. Specimen metadata, photographs, trace-files, and DNA barcode sequences were deposited in BOLD project ITLP. Complete specimen data for these individuals can be found on BOLD under the project ITLP (doi.org/10.5883/DS-ITLP). All specimens in project ITLP were intercepted in the USA. Data on assumed countries of origin was provided by APHIS-PPQ staff, based on the best information available.

### Identification using BOLD

Specimens with sequence data were analyzed using a molecular barcode approach and BOLD. To observe the growth of data in BOLD and how it can improve the ability of the system to identify specimens a comparative past and present analysis was completed. The retrospective analysis was conducted using accumulated data for the years 2009 through 2015. The current BOLD system data, as of the date this study was also used (i.e., 2019). Specimen identifications were conducted using the BOLD Species Level Barcode Record (SLBR) and the Public Record Barcode Database (PRBD) options. The BOLD system defines the SLBR option as “every COI barcode record with a species level identification and a minimum sequence length of 500bp. This includes many species represented by only one or two specimens as well as all species with interim taxonomy.” and the PRBD option as “all published COI records from BOLD and GenBank with a minimum sequence length of 500bp. This library is a collection of records from the published projects section of BOLD.”

### Concordance of identifications

The number of specimens that could be matched to a BOLD record was recorded for both the PRBD and SLBR options, across the archived and current databases (2009–2015, 2019). Concordance was measured based on taxonomic rankings obtained from BOLD. Each specimen identification was put into one of five categories for concordance: 1) a DNA identification having a match to a record (>98%) with a lower-level taxonomic name than the morphological identification, 2) a concordant identification having a match (>98%) to a record with identical species name as morphological identification, or a lower level morphological identification having a match (>98%) to a record with a higher level taxonomic name but consistent with lower level morphological identification, 3) an interim identification having a match (>98%) to a record with interim identification only (BIN) or match to a record (<98%) with the same Linnaean name or match (>98%) to a record with a Linnaean species name sister to the target species, 4) no match available using the BOLD system and the given dataset, 5) the final category was where a discordant identification result indicated a mismatch to a record in the dataset where the morphological identification and the taxonomic name associated with a record in BOLD do not agree and do not reflect a lower or higher taxonomic rank with respect to the morphological identification (>98%). A further analysis using the concordance data was conducted specifically using results from the 2019 database. This analysis was completed to determine how many specimens were identified to the species level using the current SLBR and PRBD database options versus how many were identified to the species level using morphological methods alone.

### BIN analysis

Using the 2019 SLBR database, specimens associated BINs and the records they contained evaluated. BINs were assigned to one of two categories based on the sequence’s associated taxonomic identification: 1) concordant where sequences within the BIN matched to an identical taxonomic rank or gave higher level results that were consistent with lower level taxonomic assignments or 2) discordant where taxonomic naming of sequences within this BIN did not agree.

## Results

### Sequencing

Of the 241 specimens examined, full length DNA barcodes (≥648bp) were obtained for 188 individuals. A single primer pair LepF1/LepR1 was used to generate barcodes for 173 individuals, while the remaining individuals were generated with a combination of mLepF1/LepR1 and LepF1/mLepR1. DNA Barcode fragments (≤407bp) were obtained from an additional 13 individuals, bringing the total number of specimens with sequence data to 201.

### Identification using BOLD

**Fig 1:**
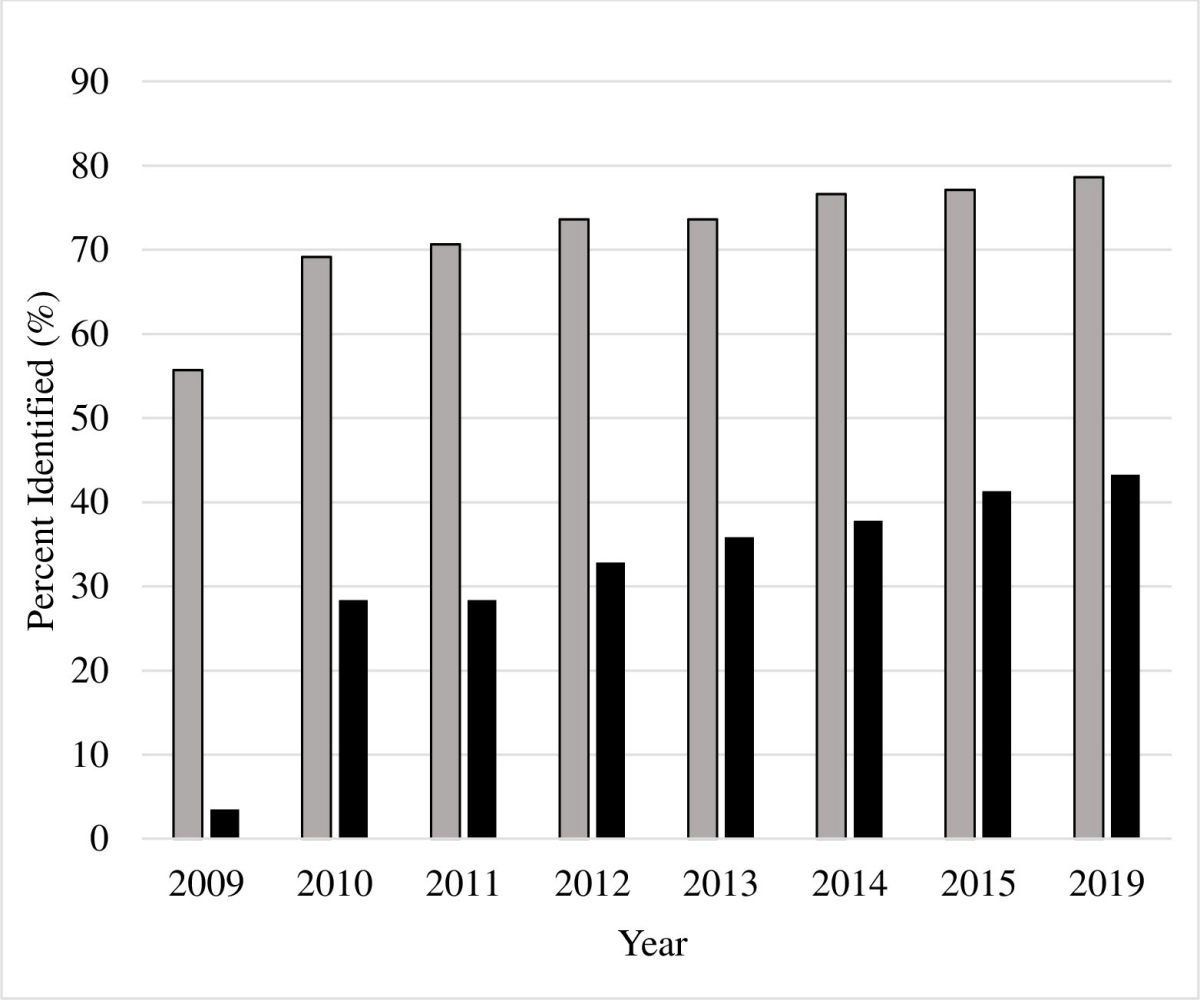
Percent identification for 201 specimen increases for SLBR and PRDB as BOLD increases in age, specimen identification by Species Level Barcode Record (SLBR = grey) and Public Record Barcode Database (PRBD = black).

For the 201 sequences, using both the SLBR (non-public) and PRDB (public) options, the number of BOLD identifications increased over time (Fig 1). Identifications by SLBR was consistently higher than identification by PRDB. Specimen identification by the 2019 PRDB option (43.2%) was unable to surpass the identification level achieved by the SLBR option in 2009 (55%). In both cases, the highest taxonomic identification was at the level of order, and the lowest taxonomic identification was at the level of species.

### Concordance of identifications

**Fig 2:**
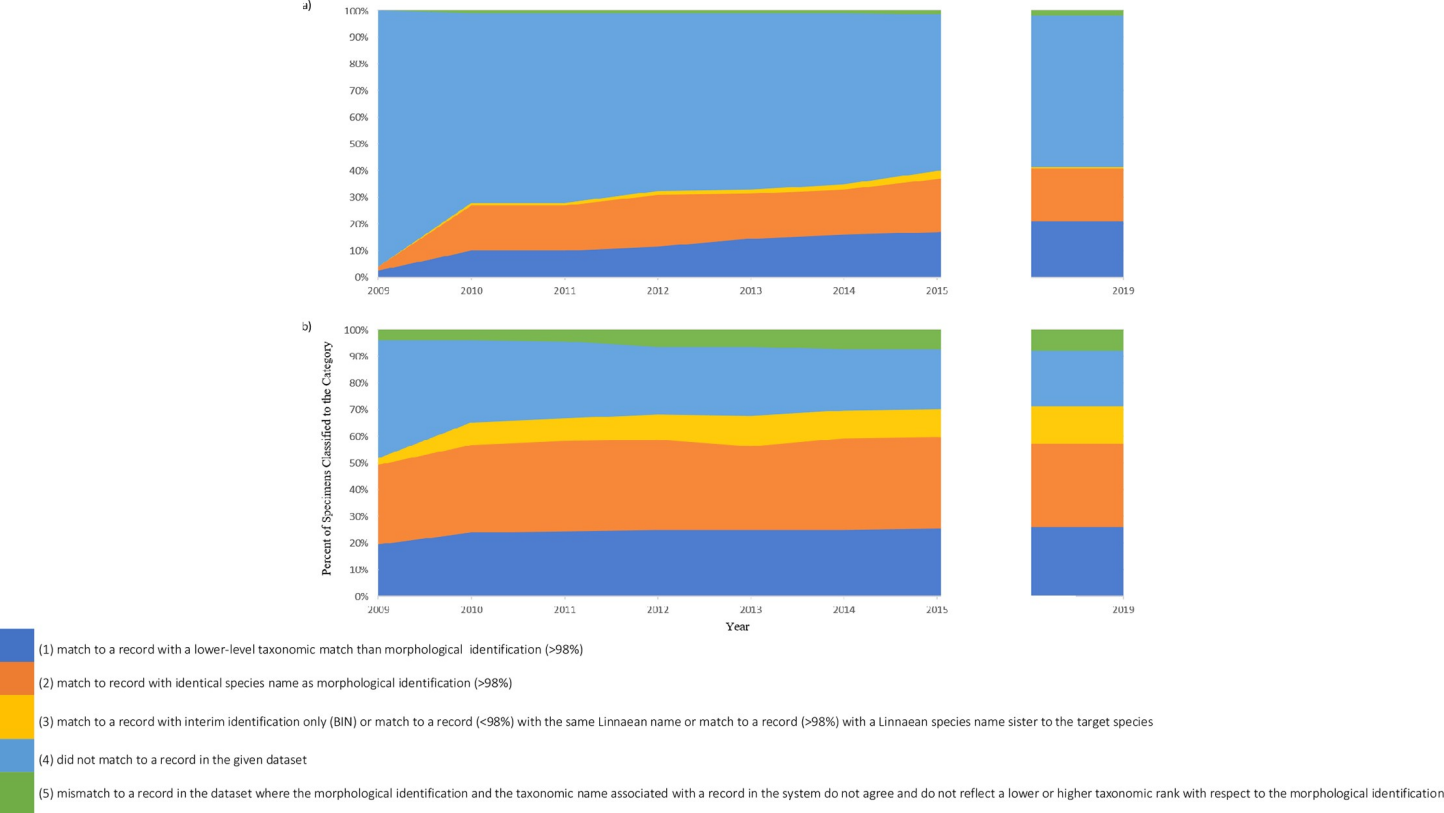
Development of concordance between the PRDB and SLBR with morphological identification over the course of 10 years, analysis of PRDB = Fig 2a, analysis of SLBR = Fig 2b.

The results of the concordance analysis are shown in Fig 2. Category 5 (specimen’s sequence did not match to a barcode record in BOLD), represented by the light-blue shaded region of Fig 2, decreased over time for both SLBR and PRDB. In the final 2019 analysis, PRDB had more specimens that matched this category as compared to SLBD identification with 56.7% and 10.9% specimens assigned to this category, respectively. For categories 1–3, all increased over time as reference libraries were being actively built. Additionally, over time there was an increase in the number of specimens assigned to category 5 (mismatch to a record in the dataset where the morphological identification and the taxonomic name associated with a record in the system do not agree and do not reflect a lower or higher taxonomic rank with respect to the morphological identification (>98%)). The increase over time in the number of specimens in category 5 was higher for the SLBD (4.0% to 7.9%) than the PRDB (0.0% to 2.0%).

A more in-depth analysis of concordance for species-level identification by morphology as compared to DNA barcoding using the 2019 PRBD and SLBR results was conducted. DNA barcoding was able to provide species-level identifications for 42.3% (85/201) of specimens using the PRBD option, and 66.7% (134/201) using the SLBR option using the 2019 dataset. This is greater than the number of specimens identified to the species level by morphology which identified only 38.3% (77/201) of the specimens to the species level. To take a closer look at concordance, the number of specimens identified to the species level by both morphology and DNA barcoding were examined. Of the 38.3% (77/201) of specimens identified to species level using morphology, 55.8% (43/77) and 100% (77/77) were also identified to the species level using the PRBD and SLBR options, respectively. Focusing on PRBD, 93.0% (40/43) gave a concordant ID, 4.65% (2/43) gave an interim-ID and 2.3% (1/43) gave a discordant ID. In contrast, using the SLBR option, 77.9% (60/77) gave a concordant ID, 14.3% (11/77) gave an interim identification and 2.6% (2/77) gave a discordant ID.

### BIN analysis

Finally, for the total set of 201 specimens, barcode analysis using the BIN system resulted in 92 BINs, 34 of which were newly established in BOLD. Upon examination, 40.2% (37/92) contained a single sequence and thus were excluded from further BIN analysis because with only a single sequence in the BIN, variation of the records corresponding to morphological identification was not possible. The remaining 59.8% (55/92) of the BINs had multiple sequences and were analyzed based on the morphological identification agreement for the records. The results indicate that in 55 BINs with multiple sequences, 85.5% (47/55) were concordant and 14.5% (8/55) were discordant. Furthermore, it was found that 61 of the BINs were based on barcode data from 10 or fewer records.

## Discussion

To demonstrate the improved utility of BOLD to identify specimens, a retrospective analysis from the years 2009 to 2015 was conducted. This was followed by an analysis of current 2019 BOLD system. To highlight a lack of data movement, we contrasted specimen identifications using public (PRDB) and non-public (SLBR) data. Concordance between morphological and molecular identifications was also studied, and the results support DNA barcoding as a valuable method for specimen identification. In addition, analysis of the BINs used in our dataset demonstrated the need to increase BOLD data for two reasons: 1) to increase our confidence in BINs that contain 10 or fewer specimens; and 2) to resolve potential species complexes demonstrated by morphological uncertainty within a single BIN. We find that the use of DNA barcoding is advantageous in identifying specimens at ports-of-entry and suggest that increased efforts are needed to continue to populate public DNA barcode record databases.

An inevitable increase in insect invasions due to globalization is expected in the coming decades, especially in developed countries that already experience a high number of invasion events [45,46]. To slow this trend, an increase in our knowledge of potential invasion pathways, and the effective storage of interception data, are necessary. In our dataset, the country of collection of the specimen was classified as “Exception Quarantine Capture” in the BOLD system. Putative origin (of the commodity) and actual point of interception (port-of-entry) are described in the BOLD “Collection Notes.” These data may provide regulatory agencies with information regarding the pathway of the commodity, thereby identifying potential points at which insect stowaways may have been acquired [45]. The use of collection localities for intercepted specimens such as the port-of-entry (e.g., “Miami, Florida” for a commodity that originated in Ghana) will leave out key information that may be valuable for the development of regulatory protocols concerning pathways of invasive species [46]. With increased globalization, clear annotation of data regarding origin versus point of interception is critical. Labelling intercepted specimens as “Exception-Quarantine Capture” provides more accuracy in the associated data and should be implemented as best practice for all future datasets concerning intercepted specimens.

As reference barcode sequences continue to be generated, bioinformatic re-examination of molecular DNA barcode data can help to evaluate progress in cataloging global biodiversity for use in molecular DNA identifications. In this study BOLD archives were used to compare specimen identifications from the years 2009–2015 to current data in 2019. Our results indicate an increase in BOLD’s ability to identify microlepidoptera specimens, for both the SLBR and PRBD data sets (Fig 1). From the years 2015 to 2019 BOLD growth for our dataset of interest slowed. These years generated a small increase in identified specimens, with the number rising by only 1.5% (SLBR) and 2.0% (PRBD). Furthermore, for the current 2019 BOLD system, identifications were achieved for only 43.3% (PRBD) and 78.6% (SLBR) of the 201 specimens in the dataset. This indicates a need for further reference library development.

The continued development of molecular libraries, like BOLD, is made possible through the support of diverse user groups with different mandates and research emphasis including biodiversity, taxonomy, and forensic applications (e.g., targeted species identifications) [33]. Continued support for discovery-based research is needed, particularly for biodiverse countries and taxa [47]. It is the initiation of cooperative regional programs for the barcoding of intercepted specimens that simultaneously allow for the confirmation of morphological identification while bolstering sequence data [48].

The data on BOLD has varying levels of access, including datasets with records that are public (PRDB) and not public (SLBR). As seen in Fig 1, the number of specimens identified by the SLBR, which includes non-public data, is much greater than those identified by the PRDB. Looking at the 2019 PRDB results only 43.3% (87/201) of specimens received an identification by BOLD. This is less than the number of specimens identified in 2009 by SLBR which was 55.7% (112/201). It is startling to see that the 2019 public database is still unable to surpass the 2009 non-public database in terms of specimen identification for our dataset. This could indicate that some data has not been made public for over 10 years. For non-public data, BOLD users do have the ability to request access to information from anonymous projects (refer to www.boldsystems.org). This gives users the potential to increase their datasets however, access may not be granted, and the process can be lengthy depending on the response time of the data-owner. Access to public data on BOLD also supplies users with metadata such as chromatogram files, the location of voucher specimens, and the presence and location of long-term storage of DNA or tissue [33, 34]. BOLD records that are public and complete with metadata are what allow for linkages between intercepted larvae and vouchered adults [33]. Alternatively, for species that are yet to be morphologically described, including a molecular approach has the potential to integrate taxonomic data, allowing specimens of different life-stages to be connected [49, 50]. It should be expected that the occurrence of linkages between specimens of two different life-stages would increase as more biodiversity-focused barcoding-library building projects are conducted [51–53].

Public access to BOLD records is also important for data verification. For DNA-based specimen identification in biosecurity programs, only records with public and complete metadata should be used. This allows for record validation by a content expert for issues that arise during the identification process. Furthermore, publicly accessible data encourages harmonization between governments, potentially leading to more efficient conflict resolution regarding regulatory blocks and other trade-related issues. This transparency may not be possible for other DNA barcode reference libraries such as Genbank [54], which historically have not supported sequence–specimen metadata associations. The adoption of systems to evaluate and/or rank the quality of specimen records [55] will be a necessary prerequisite in the widespread adaptation of DNA barcoding for regulatory applications.

The number of specimens which were matched to a record in the BOLD system has increased across the years analyzed (Fig 1). While it is true that we could expect an increase in the number of identifications due to an increase in the number of reference sequences in the database, this is not necessarily true for concordance between DNA barcoding and morphology [56–58]. Our results from Fig 2 also highlight the need for continued sampling, data acquisition, and storage in the public datasets given the large percentage of our dataset specimens which could not be identified (Fig 2 category 4), had interim identification based on molecular data (Fig 2 category 3), or could only be placed to a barcode record with a higher taxonomic rank (Fig 2 category 2). There is also need for increased data management of existing records, such as records with corresponding molecular evidence but having differing morphological taxonomic assignments (Fig 2 category 5).

There is continued need for new public data submitted to BOLD, but there is also need for the movement of existing data from the SLDB to PRDB. This movement of data will provide researchers with access to metadata necessary when evaluating the quality of a record and to verify matches. The addition of data and efforts to further curate accessible data will result in a greater number of records providing reliable sequence representation for species. In the context of invasive pest management, identifications relevant to inform biosecurity decisions must be at the species level. Obtaining specimen identifications to the species level is desired, but given the magnitude of global arthropod biodiversity, this is still challenging [49, 47]. Using the 2019 BOLD data, molecular identification surpassed morphological species assignments (made in 2009–2015) in the number of specimens that received a species-level identification; morphology 38.3% (77/201), PRDB 42.3% (85/201), and SLBR 66.7% (134/201) (Fig 2 category 1).

While DNA barcoding may not be able to provide species level identifications for all specimens at this time, its use as a complementary identification technique is recommended. Fig 2 categories 1 and 2 demonstrate that DNA barcoding is able to place a specimen to a species or taxonomic rank consistent with morphological identification for more than 30% of our cases using the PRDB 2019. These data show how the use of both DNA barcoding and morphological identifications in conjunction can provide secondary evidence to support morphological identifications and, in the category 1 cases, increase taxonomic resolution. Both outcomes are important in biosecurity as unidentifiable tissue and difficult to identify specimens (such as microlepidopterans) are often intercepted.

DNA barcoding also has value in highlighting potential misidentifications by morphological methods [59]. For this microlepidoptera dataset, DNA barcode species-level identifications lacked concordance with morphological species-level identifications for 2.3% (PRDB 2019) and 2.6% (SLBR 2019) of specimens (Fig 2 category 5). The use of record metadata, including the location of voucher specimens for re-evaluation where appropriate, is essential when scrutinizing these data to make informed decisions regarding clarification of identifications and curation of records in the database. While the cases present in this study, may represent a morphological misidentification, particularly considering the difficult nature of microlepidoptera identifications, these cases are difficult to verify due to the lack of public barcode records. Future efforts to populate the BOLD system with adult expertly identified microlepidoptera specimens is necessary to fully investigate these problematic records.

DNA barcoding can also provide interim identifications through molecular operational taxonomic units (MOTU) which, when molecular libraries are further populated, can retroactively provide identifications [49, 47]. In this dataset, intercepted specimens fell into 92 BINS. Of these, 34 BINS were new to BOLD. This is not surprising because outside of Costa Rica, there are a limited number of records of microlepidoptera with sequence data for species from the Neotropics, the origin of many of the specimens in the intercepted dataset [51]. Variation in global sample collections likely make specimen identifications biased based on region of origin. Specimen interceptions entering North America with origins from Europe and Australia are more likely to have BOLD records due to greater research efforts on microlepidoptera from these regions. In contrast, interceptions from the Neotropics, Africa, and Asia are less likely to be identified to a low taxonomic level owing to the paucity of sequences in BOLD, or their absence altogether. Due to the global nature of tracking and identifying insects of concern, a collaboration of nations, particularly those connected by shared borders or trade routes, is necessary to further build molecular barcode libraries for use in biosecurity applications [47, 60].

Nearly 15% of the BIN’s identified in our study were classified as discordant BINs (taxonomic naming of sequences within a BIN did not agree) by BOLD. This discordance may be the result of species complexes (i.e., closely related species that are not easily separated by morphology and/or barcodes) or may be the result of misidentified specimens. For species complexes, resolution relies on the associated metadata and may require additional molecular techniques, morphometry, ecology, or morphological characters from all life stages [61]. Unrecognized species complexes may be due to the use of poor taxonomic keys, inadequate sampling, identifier bias (i.e., emphasizing different morphological characters), and/or geographical naming [61]. Upon analysis of the discordant BINs by expert Lepidoptera taxonomists (co-authors Brown and Miller), some BINs were suspected as discordant due to misidentified specimens in the reference library rather than a species complex. For example, one discordant BIN (BOLD: AAP2599) contained three families; Blastobasidae, Gelechiidae and Oecophoridae, but only a single species name; *Calosima albapenella*. This use of Gelechiidae and Oecophoridae in this BIN likely represents misidentifications, with the true family being Blastobasidae. In contrast, another BIN (BOLD: AAA7690) was associated with three species, all from the same genus; *Archips packardiana*, *Archips alberta* and *Archips tsuganus*. This specific example is more likely to represent a species complex and further investigation is required to determine the source of discordance.

In the context of pest-species complexes, a higher-level taxonomic classification (i.e., genus-level identification) may be suitable to enact biosecurity actions. Therefore, clarity of BIN discordance can improve the applicability of DNA barcoding and further support biosecurity decisions. These assessments of discordant BINs required the use of accessible metadata, such as image files, and demonstrates the importance of well documented public records. Additionally, increasing sampling efforts and data accumulation typically helps reveal the cause of disagreement among records in a BIN [49]. In our dataset, 66.3% (61/92) of the BINs were comprised of less than 10 records, indicating a need to improve sampling efforts.

Material collected from biodiversity and ecological surveys provide a unique opportunity to catalogue undescribed species as well as augment distributions and sample haplotype diversity for species already represented in the reference library. It is just as important to further our efforts in obtaining expertly identified material from sources such as museum collections to increase records with species level names in the BOLD system [62]. Together, these efforts can provide a robust data system upon which the identification and subsequent verification of identifications can be conducted. DNA barcoding studies, such as plant protection and quarantine studies like this one, must endeavor to catalogue DNA barcoding data by archiving query sequences along with associated metadata. As such, it is encouraged that all query sequences be archived in publicly accessible libraries regardless of the reason they were initially generated. Through the use of MOTU approaches like BINs, the task of organizing otherwise unidentifiable material into species-like units is entirely possible on a large scale. Harmonizing these MOTUs in a central database (i.e., BOLD) greatly facilitates their use as interim taxonomy which is accessible by multiple working groups and researchers globally.

Unfortunately, 16.6% (40/241) of intercepted specimens in this dataset did not yield sequence data, which was surprising given that most samples were less than one year old and presumably experienced similar storage conditions. Considering that the primers employed in this study have been used extensively for barcoding of Lepidoptera [44, 51, 52, 63–65], it seems likely that failure to amplify sequences from these specimens was a result of DNA quality rather than primer selection. One explanation is that larval samples from APHIS-PPQ are typically submitted in 75% ethanol, which may result in the degradation of DNA over time. In addition to data collection, we would encourage the use of storage methods amenable to molecular data acquisition such as the storage of specimens or tissue in >95% ethanol along with the immediate and consistent storage of ethanol preserved specimens at low temperatures until genetic material is obtained [66]. These efforts can dramatically increase our ability to obtain molecular data from specimens and accomplish many of the advantages discussed above.

## Conclusion

The identification of specimens for biosecurity are time sensitive; i.e., they are most valuable when made within a few hours, not days. Although barcoding may increase the reliability of “routine” identifications (i.e., those submitted without an associated deadline), as of yet, the process is unable to assist in the identification of specimens submitted as “urgent” (i.e., requiring rapid turnaround), where morphological identification remains the best option. Even so, increasing the accuracy of “routine” identifications can help to speed the DNA barcoding of specimens, making future rapid identifications using a molecular approach feasible. This study demonstrates the need for the accumulation of records relevant to biosurveillance globally. Furthermore, recording location data for suspected country of origin and country of interception separately, needs to be standardized as the method of reporting data for border-interceptions. With increased globalization the use of both morphological and molecular identification approaches is necessary if we want to effectively combat the growing number of intercepted specimens at ports-of-entry. A combined approach is essential for building DNA barcoding reference libraries thereby increasing the reliability of identifications which will inform future biosecurity actions.
